# The Coexistence of Systemic Lupus Erythematosus and Thyrotoxicosis: The Diagnostic Value of Antihistone Antibodies

**DOI:** 10.1155/2012/517059

**Published:** 2012-04-09

**Authors:** Marta Baleva, Krasimir Nikolov, Emil Manov, Anastas Stoikov, Rebhat Shabani, Lyubomir Dourmishev, Milena Nikolova

**Affiliations:** ^1^Department of Clinical Immunology, University Hospital Alexandrovska, Sofia, Bulgaria; ^2^Department of Dermatology and Venereology, Medical University, 9002 Varna, Bulgaria; ^3^Department of Internal Medicine II, University Hospital Alexandrovska, Sofia, Bulgaria; ^4^Department of Dermatology and Venereology, University Hospital Alexandrovska, Sofia, Bulgaria; ^5^Department of Nephrology, University Hospital Alexandrovska, Sofia, Bulgaria

## Abstract

We report four female patients with Graves' disease with positive ANA antibodies and possibility for development of systemic lupus erythematosus. All four patients have been treated with antithyroid drugs. SLE symptoms have appeared from 4 to 12 months after the beginning of therapy with methysol in two of them. The third patient had no symptoms for SLE, but her ANA, anti-DNA, and antihistone antibodies had been positive at the time of the onset of thyrotoxicosis. The fourth patient had alopecia areata with positive ANA and antihistone antibodies.

## 1. Introduction

The association between Hashimoto's thyroiditis and systemic lupus erythematosus (SLE) has been described by a number of authors [1, 2], but in contrast Mulhern et al. [3] report that there is not clear association between these two diseases. The coexistence of SLE and thyrotoxicosis was less known till now. In some cases thyrotoxicosis preceded the manifestation of SLE, in other ones the signs of SLE appeared firstly, and in some patients both diseases began simultaneously [4].

We describe four patients with Graves' disease with positive antinuclear antibodies (ANAs) and a possibility for development of SLE.

ANAs were determined by an indirect immunofluorescent method on HEp-2 cells, anti-ds DNA, anti-Sm, anni-RNP, and antihistone antibodies, as well as T_3_, T_4_, and TTH—by an ELISA technique.

## 2. Case Report

**Patient 1**

A 39-year-old female patient had suffered from weakness, weight loss, tachyarrhythmia, tremor, exophthalmos, moist skin, and goiter during the last year. For four months she was treated with propylthiouracil (PTU) with no effect, but one month ago she had edema on the legs, pleural effusion, and dyspnea. At the time of hospitalization in the intensive care department, she had exophthalmus, tachyarrhythmia (140 bpm), and anasarca with no effect of treatment with PTU 60 mg daily. Leading clinical manifestations were signs and symptoms for cardiac decompensation with cardiomegaly, absolute tachyarrhythmia with atrial flutter, T_3_ gallop rhythm, positive venous pulse of the jugular veins, hypotension, and anasarca. Her skin and mucus were icteric; she had xanthelasmas on the upper eyelids and goiter (III-IV grade) with an auscultatory thrill, and the pleural effusion and the hepatomegaly persisted. Laboratory tests showed normal values of ESR, RBC, WBC, and platelet counts and of serum creatinine, urea, and ASAT and ALAT levels, bilirubinemia (total—48, 3 *μ*mol/L, direct—17, 3 *μ*mol/L), elevated levels of cholestatic enzymes (alkaline phosphatase 168 Units, gamma-GTP 62 Units), negative ANA, DNA, Sm, and RNP, but positive antihistone antibodies, T_3_-3, T_4_-124, and TTH-0, 1 pmol/L. 12-lead ECG: absolute tachyarrhythmia and atrial flutter, P-pulmonale, diffuse changes in repolarisation showing positive results after therapy. Echocardiography: left ventricle sizes and volumes are within the normal range, and moderate enlargement of the left atrium exists. Elevated pulmonary artery pressure (systolic: 60 mm Hg, median 38 mm Hg) and overburdened right ventricle with relative tricuspidal regurgitation II degree and enlargement of the right atrium were detected. EF was 44%, and FS was 22%. She was treated with methylprednisolone 30 mg daily and furosemide 40 mg daily and showed improvement.

**Patient 2**

The case is a 44-year-old female patient, who had suffered from tachycardia, moist skin, and goiter in the last years. She had pericardial effusionseveral months ago. At that time the values of T_3_ and T_4_ were 8 and 115 pmol/L, respectively. The patient was treated with methysol (6 tablets daily, later 4 tablets daily). Since last summer she had had photosensitivity, arthralgia, alopecia areata, and malar rush. ANAs were positive (1 : 640), and DNA and Sm antibodies were positive as well, but antihistone antibodies were negative. All blood tests, including ESR, erythrocytes, leukocytes, thrombocytes, ASAT, ALAT, creatinine, and urea, were normal. The levels of thyroid hormones were as followes: FT_3_ = 5.3 pg/mL (normal 2.3–4.2 pg/mL); FT_4_ = 9.3 *μ*g/dL (normal 0.8–1.8 ng/L), TSH-below 0.15 *μ*IU/mL (normal 0.5–4.70 *μ*IU/mL). Arthralgia, photosensitivity, alopecia, and malar rash persist till now. In addition this patient had WPW syndrome and allergy to antibiotics.

The patient was treated with methysol, but because of an elevation of the serum transaminases levels during the course of treatment (ASAT-37, ALAT-64 U) and hyperbilirubinemia, the treatment with methysol was stopped and PTU (3 tablets daily) was given. Additionally she took syllimarine (6 tablets daily) and chloroquine (1 tablet).

**Patient 3**

A 29-year-old female patient was admitted to the hospital because of tachycardia (100 bpm), tremor, weakness, edema on the upper eyelids and legs, skin itching, and enlarged goiter. Tc 99 m thyroid scan revealed increased uptake and uneven distribution of radioactivity. EST, hemoglobin, erythrocytes, leukocytes, thrombocytes, creatinine, and urea were normal, and the following results were obtained: ASAT = 55 IU/L, ALAT = 58 IU/L, alkaline phosphatase = 138 IU/L, FT_3_ = 11, FT_4_ = 60, anti-DNA and antihistone antibodies-positive, Sm, RNP-negative. The patient was treated with methysol 2 tablets 3 times daily, propranolol 60 mg daily, and Hismanal.

**Patient 4**

A 29-year-old female patient had suffered from thyrotoxicosis for the last 15 years. The first symptoms were weakness, tremor, tachycardia, diffuse goiter, exophthalmus, and alopecia areata. She had been treated with thymidazol, propranolol, and medazepam for the following 3 years. Alopecia disappeared, and symptoms for thyrotoxicosis showed an improvement. Ten years ago she had alopecia areata again. The treatment with low doses of prednisolone (20 mg daily) and laser therapy was followed by episodical disappearance of the alopecia. At the admission to hospital, her T_3_ was 6 mmol/L, T_4_-9, 5 mmol/L and the clinical symptoms for thyrotoxicosis were negative, but she had reticular alopecia areata. ANAs were positive (1 : 160), while Sm, RNP, and DNA antibodies were negative, but antihistone antibodies were positive.

All clinical and laboratory data are summarized in Table 1.

## 3. Discussion

Graves' disease and SLE are multisystemic autoimmune disorders. It is well known that many patients with SLE have positive thyroglobulin and microsome autoantibodies [1, 5, 6], and they have higher frequency of thyroid disorders [4, 7, 8]. Some authors suggest that the existence of Graves' disease induced SLE. For example Searles et al. [9], Amrhein et al. [10], Sato-Matsumura et al. [11], and Horton et al. [12] discuss the existence of PTU-induced lupus-like syndrome. The coexistence of thyrotoxicosis and SLE has been described by others [4, 13, 14]. According to Rodrigue et al. [4], the onset of hyperthyroidism preceded by 6 months to five years the onset of lupus manifestations in 3 from 6 patients; in 2 patients both diseases began simultaneously, and in the last patient the diagnosis of SLE preceded that of thyrotoxicosis.

In 2 of four patients described in this paper the SLE signs and symptoms follow those of thyrotoxicosis for a period of 4 and 12 months (patient 1 and patient 2, resp.). In the third patient, positive ANA appeared simultaneously with the signs and symptoms of thyrotoxicosis. The fourth patient had alopecia areata at the onset of thyrotoxicosis, but her ANAs were not determined at the beginning of the disease. At the time of observation, she had alopecia areata and positive ANA and antihistone antibodies. All four patients have been treated with PTU, methysol, and thymidazol for a typical Graves' disease: goiter, exophthalmas, tremor, tachycardia, and changes in thyroid function. Two of them had some symptoms characteristic for SLE: polyserositis and antihistone antibodies (N 1) and arthralgia, alopecia, photosensitivity, malar rush, and positive ANA, DNA, and Sm antibodies (N 2). The third patient had positive ANA, anti-DNA and antihistone antibodies, and no clinical data for SLE. The fourth one had only alopecia areata and photosensitivity as symptoms of SLE; ANA titer was elevated, and antihistone antibodies were positive.

In 1992 Loviselli et al. [15] reported that 13% of the patients with Graves' disease are positive for ds-DNA antibodies, determined by RIA, 11% for ss-DNA antibodies (ELISA), 2% for antihistone antibodies (ELISA), and 7% for ANA (immunofluorescence). Park et al. [6] do not find any significant differences in the concentration of anti-DNA antibodies in SLE patients with normal thyroid function, Graves' disease, Hashimoto's thyroiditis, and euthyroid sick syndrome. All 6 patients with coexistence of SLE and thyrotoxicosis, described by Rodrigue et al. [4] are ANA positive and 4 of them anti-DNA antibodies positive. Krausz et al. [16] report a patient in whom SLE has preceded Graves' disease for several years, and after treatment with methimazole, an exacerbation of SLE activity has been observed as well as an elevation of ANA titer.

Recently there are at least 4 opinions about the relations between SLE and thyrotoxicosis:

1. the coexistence of SLE and thyrotoxicosis [4, 17];
2. the possibility of drug-induced SLE after treatment with antithyroid drugs [10, 12];
3. the presence of autoimmune thyroid disorders in SLE patients [5, 8, 18];
4. the possibility of drug-induced serological changes after treatment with propylthiouracil (positive ANA of different type without presence of SLE) [19, 20].

The four patients examined by us have different antinuclear antibodies (Table 1), but only one of them (N 2) fulfills the ARA criteria for SLE. All four patients have been treated with antithyroid drugs, but 3 of them have positive antihistone antibodies. SLE symptoms have appeared 12 months after the beginning of methysol therapy in the second patient and the polyserositis in the first patient 4 months after the beginning of therapy with PTU.

We share the opinion of Rodrigue et al. [4] that the early differential diagnosis between SLE and thyrotoxicosis is very difficult. The reasons for this are some similar clinical manifestations of both diseases. Another explanation is the possibility of drug-induced SLE in some patients, treated with antithyroid drugs. The determination of ANA, including antihistone antibodies, could be of great interest in these cases.

## Figures and Tables

**Table 1:** Clinical symptoms and laboratory data in four patients with thyrotoxicosis.

*N*	Clinical symptoms for thyrotoxicosis	T_3_/FT_3_	T_4_/FT_4_	Clinical symptoms for SLE	ANA	Sm Ab*	RNP Ab	ds DNA Ab	histone Ab
1	Diffuse goiter, exophthalmus, tachycardia, moist skin, weight loss	6.8	183	Polyserositis	—	−	−	−	+
2	Diffuse goiter, tachycardia, moist skin	8	115	Pericarditis, photosensitivity, arthralgia, alopecia areata, malar rash	1 : 640	+	−	+	−
3	Tachycardia, tremor, diffuse goiter, weakness	11	60	No	1 : 160	−	−	+	+
4	Weakness, tachycardia, diffuse goiter, tremor, exophthalmus	6	9.5	Alopecia areata, photosensitivity	1 : 160	−	−	−	+

*Ab: antibodies.
